# Heuristic Approaches for Enhancing the Privacy of the Leader in IoT Networks

**DOI:** 10.3390/s19183886

**Published:** 2019-09-09

**Authors:** Jie Ji, Guohua Wu, Jinguo Shuai, Zhen Zhang, Zhen Wang, Yizhi Ren

**Affiliations:** School of Cyberspace, Hangzhou Dianzi University, Hangzhou 310018, Zhejiang, China

**Keywords:** Internet of Things, network analysis, closeness centrality, greedy algorithm, optimization

## Abstract

The privacy and security of the Internet of Things (IoT) are emerging as popular issues in the IoT. At present, there exist several pieces of research on network analysis on the IoT network, and malicious network analysis may threaten the privacy and security of the leader in the IoT networks. With this in mind, we focus on how to avoid malicious network analysis by modifying the topology of the IoT network and we choose closeness centrality as the network analysis tool. This paper makes three key contributions toward this problem: (1) An optimization problem of removing *k* edges to minimize (maximize) the closeness value (rank) of the leader; (2) A greedy (greedy and simulated annealing) algorithm to solve the closeness value (rank) case of the proposed optimization problem in polynomial time; and (3)UpdateCloseness (FastTopRank)—algorithm for computing closeness value (rank) efficiently. Experimental results prove the efficiency of our pruning algorithms and show that our heuristic algorithms can obtain accurate solutions compared with the optimal solution (the approximation ratio in the worst case is 0.85) and outperform the solutions obtained by other baseline algorithms (e.g., choose *k* edges with the highest degree sum).

## 1. Introduction

### 1.1. Background

In recent years, centrality analysis, a kind of network analysis tool, has been applied in the area of Internet of Things (IoT) and can be used to analyze the topology of the IoT network. For example, closeness centrality can be chosen as the measurement for IoT device selection by identifying the central nodes in the dynamic IoT network [1]. However, if attackers use centrality analysis to find the important nodes in the IoT network, they can launch more accurate attacks such as DDoS attacks or deceive the important nodes to spread fraudulent information in the network [2]. As a consequence, defensive strategies against malicious centrality analysis are especially necessary, i.e., the question turns into “How to avoid being detected by malicious centrality analysis?”.

As far as we know, few researchers have focused on this question in the area of Internet of Things (IoT) and the research most relevant to this question was proposed by Waniek [3]. Inspired by Waniek’s work, our work aims at helping the **leader** in the IoT network avoid being detected by malicious closeness analysis. **Closeness centrality** [4] is chosen as the measurement of the importance of the nodes in the IoT network and **leader** (the protected target of our work) is the node that has the highest closeness value in the network. The **leader** often has the greatest impact on other nodes or has the clearest understanding of the network [5] and it is vulnerable to be analyzed and attacked by attackers.

Against this background, to guarantee individual privacy and cyber security, we attempt to solve the above problem by removing limited links in the network to help the **leader** not be found by the attackers who use the closeness centrality analysis. Two cases of closeness value and rank are concerned. In both cases, we assume that the attacker finds the **leader** node by top-*k* algorithm and in the closeness value case the **leader** node does not know the exact value of *k* and has to minimize its closeness value. To evade attacker’s analysis, the **leader** needs to remove limited links in the network (guaranteeing the connectivity of the network) and minimize (maximize) its closeness value (rank). In our work, formally, the above problem is considered to be an optimization problem: “ How to minimize (maximize) the closeness value (rank) of the **leader** by removing *k* edges ? ”

### 1.2. Our Contributions

This paper is the expansion of the shorter conference version presented at the 5th International Symposium on Security and Privacy in Social Networks and Big Data. The contributions of our initial conference paper show as follows:

- An optimization problem of removing *k* edges to minimize the closeness value of the **leader** and its complexity analysis.
- A **greedy** algorithm to solve the proposed optimization problem in polynomial time and theoretical proof of the lower bound of its solution.
- An effective pruning algorithm—**UpdateCloseness** for computing closeness value after removing an edge.
- Experimental evaluation of the efficiency and accuracy of the proposed algorithms.

In this paper, we extend the optimization problem proposed in the conference paper to the closeness rank case. The contributions of this paper show as follows:

- An optimization problem of removing *k* edges to maximize the closeness rank of the **leader** and its complexity analysis.
- An approximation algorithm (**GSA**) combing **greedy** algorithm and **simulated annealing** algorithm to solve the proposed optimization problem in polynomial time.
- An effective pruning algorithm—**FastTopRank** for computing closeness rank of the high ranking nodes.
- Experimental evaluation of the efficiency and accuracy of the proposed algorithms.

## 2. Preliminaries

### 2.1. Basic Notation

Let G=(V,E) be a network which is a simple undirected graph, and which has n:=|V| nodes and m:=|E| edges. The edge with u,v∈V is denoted as (u,v). For the node *u*, N(u) denotes the neighbors of *u*, i.e., N(u)={v|(u,v)∈E}. For nodes *u* and *v*, P={u,…,v} denotes the shortest path between *u* and *v* and $d_{uv}$ denotes the distance of the shortest path *P*.

Given a set of edges R⊂E, G(R) denotes the subgraph after removing the set of edges *R* in *G*, i.e., G(R)=(V,E/R). Also, after removing a set of edges *R*, the shortest-path distance between node *u* and node *v* can be denoted as $d_{uv}\left(R\right)$.

**Closeness centrality** was proposed by Beauchamp [4]. This measurement quantifies the importance of a given node according to the shortest-path distances from the given node to all other nodes and requires the connectivity of the network. For a given node *u*, the normalized **closeness centrality**$c_{u}$ is defined as follows:

(1) $$c_{u}=\frac{n-1}{\sum_{v\in V\backslash \{u\}}d_{uv}}$$

The closeness rank of the node *u* is denoted as $r_{u}$ and it is possible that the closeness values of two nodes are equal. To measure the rank of them, standard competition ranking (“1224” ranking) is used in this paper.

### 2.2. Related Work

#### 2.2.1. Closeness Algorithm

In tradition schemes, the closeness value of s node can be computed by running a breadth-first search (BFS) in the network and it requires O(n+m) time. Therefore, it requires O(n(n+m)) to obtain the closeness rank of the node by computing all closeness values of each nodes. Obviously, the IoT network is a kind of complex network and the traditional way to compute the closeness value and rank is infeasible in our work. The related works of closeness computation algorithm of our work are as follows:

Top-*k* closeness and approximate closeness rank: In real-life scenarios, people pay more attention to identifying top-*k* nodes in the network or the rank of a node than the closeness value of a node. Okamoto [6] proposed the first study of top-*k* closeness algorithm and several works are proposed to improve this algorithm [7,8,9,10]. Saxena [11] first proposed closeness rank approximation algorithm by sigmoid curve and the time complexity is O(m). Bisenius [12] first proposed a dynamic top-*k* closeness algorithm, i.e., computing the top-*k* nodes after removing or adding an edge.

Dynamic update closeness: To compute the closeness value in the dynamic network, several pieces of research try to update value or rank after edge deletion and addition. Tong [13] analyzed the characteristics of edge deletion and addition on centrality and proposed a scalable and efficient algorithm to find the edges that help information propagation in the network. Santos [14] proposed an approximation closeness value algorithm that can be used after edge deletion. Sarıyüce [15], Kas [16] and Yen [17] proposed their novel closeness value update algorithm in the dynamic network.

The pruning algorithms **UpdateCloseness** and **FastTopRank** are inspired by Yen’s work [17] and Bergamini’s work [7] mentioned above, respectively.

#### 2.2.2. Topic

To the best of our knowledge, there are two works whose topics are closely related to our work. Table 1 shows the comparison between our work with the existing related works. Our work differs from [3,19] in several aspects as follows:

- Please note that [3,19] focused on the optimization of the value after updating edges. In a further step, we propose methods that can achieve the optimization of the closeness value and rank simultaneously.
- As shown in Rochat’s work [21], harmonic centrality only performs a little better in the unconnected network. However, usually the Internet of Things needs to be connected. Hence, differing from Crescenzi’s work [19], we choose **closeness centrality** as the measurement of identifying the importance of a node.
- We extend the selection range of the removing edges from the neighbors of the target node to the entire network despite the extra time cost.

## 3. Problem Definition

### 3.1. Theoretical Definition

In this section, we propose the basic theoretical definitions of the optimization problems mentioned in Section 1 and analyze the complexity of the problems. We choose the node with the highest closeness (also called **leader**) and attempt to minimize (maximize) its closeness value (rank) by removing limited edges.

**Definition** **1** **(Leader** **Closeness** **Value** **Minimization** **Problem)** *The problem is defined by a tuple (G,u,R,k). Let G=(V,E) be a network which is unweighted, undirected and connected, u∈V is **leader** with the maximum closeness value, R⊂E is the set of edges that to be removed, k∈Z denotes the maximum of the edges to be removed. The problem is to find a set of edges, R⊂E and |R|≤k, and G(R)=(V,E\R) is connected, and R is in:*

(2) $$\underset{R\subset E,|R|\leq k}{\arg\min}\;c_{u}\left(G\left(R\right)\right).$$

**Definition** **2** **(Leader** **Closeness** **Rank** **Maximization** **Problem)**  *The problem is defined by a tuple (G,u,R,k). Let G=(V,E) be a network which is unweighted, undirected and connected, u∈V is **leader** with the maximum closeness value, R⊂E is the set of edges that to be removed, k∈Z denotes the maximum of the edges to be removed. The problem is to find a set of edges, R⊂E and |R|≤k, and G(R)=(V,E\R) is connected, and R is in:*

(3) $$\underset{R\subset E,|R|\leq k}{\arg\max}\;r_{u}\left(G\left(R\right)\right).$$

In the problem LCVMIN and LCRMAX, we assume that the modified network G(R) is still connected after removing the edges of set *R* to remain the integrity of the network. We consider a budget *k* to limit the negative effects of removing edges on the network. For ease in explanation, the networks in our work are **undirected unweighted**.

### 3.2. Complexity Analysis

In this section, we study the optimization problems from the computational point of view. We prove the NP-hard of the LCVMIN and LCRMAX problem by Theorem 1 and 2 as follows. In the following Theorem 1 and 2, we will make use of the Hamiltonian cycle problem to prove that LCVMIN and LCRMAX problem cannot be solved by a polynomial-time scheme unless P=NP and these two problems are NP-hard.

**Theorem** **1.** *Leader Closeness Value Minimization Problem is NP-hard.*

**Proof.** **Reduction** **from** **Hamiltonian** **cycle** **problem** **to** **the** **LCVMIN** **problem:**  First, we propose the decision version of the LCVMIN problem: given a connected and undirected network G=(V,E), the **leader***u*, a budget k∈Z and a value x∈R, does there exist a set of edges to be removed *R* such that R⊂E,|R|≤k and the modified network G(R) is still connected and $c_{u}\left(R\right)\leq x$?

**Figure 1:**
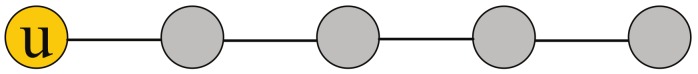
The minimum (maximum) case of closeness value (rank).

To prove the decision version problem is NP-hard, the possible smallest closeness value of the node *m* in the connected and undirected network is denoted as t∈N and we find that $m=\frac{n-1}{\sum_{i=1}^{n-1}i}=\frac{2}{n}$ for the case that the **leader***u* is the end of a path (Figure 1). This kind of network can be denoted as *M* and the closeness value of the **leader** *u* is *m*, i.e., $c_{u}=\frac{2}{n}$. Hence, we select an arbitrary instance of the Hamiltonian cycle problem (i.e., whether there is a cycle through the network that visits each node exactly once) and convert it into an arbitrary instance of the decision version of the LCVMIN problem, such that reduce the closeness value of the **leader** to a value smaller than or equal to *m*.

Given an arbitrary network G=(V,E) (undirected and connected), we will show that if G has a Hamiltonian cycle then it is possible to obtain *M* by removing |E|−|V|+1 edges as follows:

- At first, we choose a set of edges $R^{*}=\left\{e_{1},\ldots ,e_{m}\right\}$, and $|R^{*}\left|=m=|E|-|V\right|$. After removing the set of edges $R^{*}$, there is a Hamiltonian cycle in the modified network $G^{*}=\left(V,E\backslash R^{*}\right)=\left(V,E^{*}\right)$.
- Secondly, for the **leader** *u* in the Hamiltonian cycle, there are two edges $\left(u,w\right),\left(u,v\right)\in E^{*}$ and after removing one of these two edges, the target network *M* is obtained and the closeness value of the **leader***u*, $c_{u}=\frac{2}{n}$.

**Figure 2:**
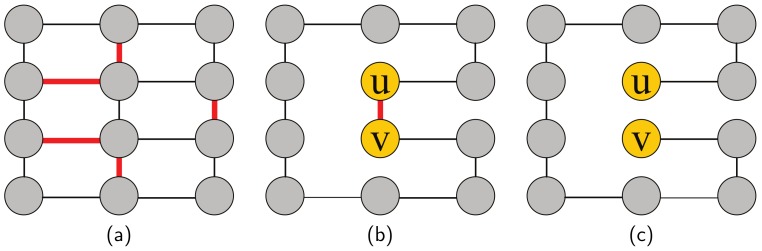
The specific steps of the reduction from the Hamiltonian cycle problem to the LCVMIN and LCRMAX problem: (**a**) original sample network, (**b**) modified network with a Hamiltonian cycle, (**c**) modified network with a Hamiltonian path.

We have proposed the procedure of the reduction above and will present an example of the reduction. We use bold red line to represent the deleted edge (Figure 2a). After removing all bold red lines, there exists a Hamiltonian cycle in the graph (Figure 2b) and in Figure 2c we can find a Hamiltonian path from *u* to *v*, which is the minimum case of the closeness of the *leader* node *u*. Therefore, we have proved that the LCVMIN problem is NP-hard by this reduction. □

**Theorem** **2.**  *Leader Closeness Rank Maximization Problem is NP-hard.*

****Proof.** **Reduction** **from** **Hamiltonian** **cycle** **problem** **to** **the** **LCRMAX** **problem:****  Similar to Theorem 1, we can find that the node *u* in Figure 1 also has the maximum closeness rank, i.e., we can make *leader* the last in the graph by the reduction from Hamiltonian cycle problem to the LCRMAX problem. Therefore, we can prove that the LCRMAX problem is NP-hard by this reduction and a simple example is shown in Figure 2. □

## 4. Approach

In Section 3, we define the LCVMIN and LCRMAX optimization problem and prove the NP-hard of the LCVMIN and LCRMAX problem. To solve these two optimization problems in real-life scenarios, in this section, we design two approximation algorithms to find the set of edges to be removed to minimize (maximize) leader node’s closeness value (rank) in polynomial time and design two pruning algorithms to compute the closeness value (rank) in limited time.

### 4.1. Approximation Algorithm for LCVMIN Problem

#### 4.1.1. Greedy Algorithm

In this section, we consider a greedy algorithm to obtain an approximate solution of the optimization problem(LCVMIN) in polynomial time, and the detail of the original greedy algorithm is shown in Algorithm 1. Our greedy algorithm attempts to find the edge e∈E which minimizes the closeness value of the **leader** at each iteration (Line 3–7). Despite that greedy algorithm can obtain solutions in polynomial time, we have to run BFS ( breadth-first search ) to compute the closeness value after removing an edge at line 5 in Algorithm 1, which requires O(n+m) and is infeasible in real-life complex networks. Therefore, in our work, we provide a pruning algorithm to reduce the number of traversed edges and nodes when recomputing the **leader**’s closeness value after removing an edge. We name it as **UpdateCloseness** algorithm and this pruning algorithm is inspired by Yen’s work [17].
**Algorithm 1:** GreedyReduction.
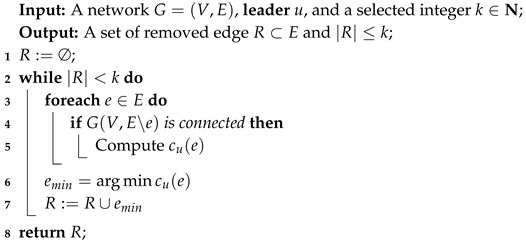


#### 4.1.2. The Approximation Ratio of the Greedy Algorithm

In this section, we prove that the greedy algorithm can offer an approximate solution to the LCVMIN problem while the greedy solution can have a ($1-\frac{1}{e}$)-approximation ratio. To complete this proof, the shortest-path sum function is chosen as the objective function of this proof, not the closeness computation function. In other words, the LCVMIN problem has been converted into the shortest-path sum maximization problem and the greedy solutions to this problem can obtain a ($1-\frac{1}{e}$)-approximation ratio by proving its **monotone** and **submodular** [22]. The detail is shown in Theorem 3.

**Theorem** **3.**  *For the* ***leader*** node u, let $R^{*}$ be the optimal solution of the LCVMIN problem, and let $R^{{}^{\prime }}$ be the solution obtained by greedy algorithm, and given the shortest-path sum function $f\left(R\right)=\sum_{v\in V\backslash \{u\}}d_{uv}\left(R\right)$. Then $\frac{f(R^{{}^{\prime }})}{f(R^{*})}=\frac{\sum_{v\in V\backslash \{u\}}d_{uv}\left(R^{{}^{\prime }}\right)}{\sum_{v\in V\backslash \{u\}}d_{uv}\left(R^{*}\right)}\geq 1-\frac{1}{e}.$

**Proof.**  We assume that the connectivity of the network cannot be influenced by edge deletion. First, for the given network G=(V,E), we can observe that the shortest-path distance between the **leader** node *u* and the other node *t*, $d_{ut}\left(E\backslash e\right)$, cannot be decreased by edge deletion, i.e., $d_{ut}\left(E\backslash e\right)\geq d_{ut}\left(E\right)$. Therefore we find that for any subset of solutions for LCVMIN problem A⊆R, f(A∪e)≥f(A) for all edges e∈R−A where *R* is the solution set for LCVMIN problem. We prove that the shortest-path sum function *f* is **monotone**.

Second, we assume that there are two solutions for LCVMIN, **A** and **B**, and A⊆B⊆R. For each edge e∈R−B, we should prove that

(4) f(A∪e)−f(A)≥f(B∪e)−f(B)

Or another form of expression as follows:

(5) $$\sum_{v\in V\backslash \{u\}}d_{uv}\left(A\cup e\right)-\sum_{v\in V\backslash \{u\}}d_{uv}\left(A\right)\geq \sum_{v\in V\backslash \{u\}}d_{uv}\left(B\cup e\right)-\sum_{v\in V\backslash \{u\}}d_{uv}\left(B\right)$$

To prove the inequality (5), we discuss two possible cases as follows:

(1) We assume that all the shortest path distances between the target node and the other nodes remain constant after removing an edge e, i.e., $\sum_{v\in V\backslash \{u\}}d_{uv}\left(A\cup e\right)-\sum_{v\in V\backslash \{u\}}d_{uv}\left(A\right)=0$. In this case, we must prove that

(6) $$0\geq \sum_{v\in V\backslash \{u\}}d_{uv}\left(B\cup e\right)-\sum_{v\in V\backslash \{u\}}d_{uv}\left(B\right)$$

this inequation can only hold when $\sum_{v\in V\backslash \{u\}}d_{uv}\left(B\cup e\right)=\sum_{v\in V\backslash \{u\}}d_{uv}\left(B\right)$ because *f* is **monotone** which has been proved above.

(2) We assume a set of edges C=R−B and $\sum_{v\in V\backslash \{u\}}d_{uv}\left(B\cup C\right)=\sum_{v\in V\backslash \{u\}}d_{uv}\left(R\right)$. Hence, we must prove that

(7) $$\sum_{v\in V\backslash \{u\}}d_{uv}\left(B\right)-\sum_{v\in V\backslash \{u\}}d_{uv}\left(A\right)>\sum_{v\in V\backslash \{u\}}d_{uv}\left(A\cup C\right)-\sum_{v\in V\backslash \{u\}}d_{uv}\left(R\right)$$

In inequation (7), $\sum_{v\in V\backslash \{u\}}d_{uv}\left(B\right)-\sum_{v\in V\backslash \{u\}}d_{uv}\left(A\right)>0$ because *f* is **monotone**. Meanwhile, A∪C⊂R and therefore, by the monotone of the function, $\sum_{v\in V\backslash \{u\}}d_{uv}\left(A\cup C\right)-\sum_{v\in V\backslash \{u\}}d_{uv}\left(R\right)<0$. Hence, the inequation (7) is proved. Now, we have proved inequation (5) by the above two cases and hence, the **submodular** of the shortest-path sum function *f* is proved.

Considering the optimal solution for the LCVMIN problem $R^{*}$ and let $R^{{}^{\prime }}$ be the solution obtained by the greedy algorithm, according to the Nemhauser’s work [22], we can prove that:

(8) $$f\left(R^{{}^{\prime }}\right)\geq \left(1-\frac{1}{e}\right)f\left(R^{*}\right)$$

Thus, Theorem 3 is proved. □

**Corollary** **1.** *The LCVMIN problem subjected to a cardinality constraint admits a $1-\frac{1}{e}$ approximation algorithm.*

Therefore, we have exploited that the greedy approximation algorithm can obtain a solution of the LCVMIN optimization problem in a lower bound $1-\frac{1}{e}\approx 0.63$. Furthermore, in Section 5, we show that the solution obtained by the greedy approximation algorithm can be more accurate than this lower bound.

#### 4.1.3. Example of the UpdateCloseness Algorithm

**Figure 3:**
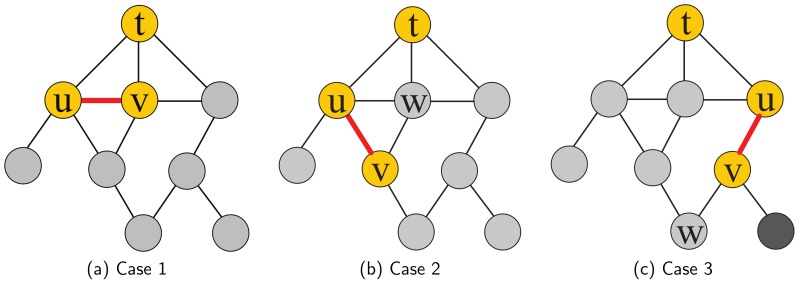
Three cases of the edge deletion.

The goal of our **UpdateCloseness** algorithm is to update the shortest paths of the nodes which is affected by removing an edge and avoid recomputing closeness value by traversing the entire network. In this section, we propose a simple example to illustrate the principle of our **UpdateCloseness** algorithm. Suppose that we have a network G=(V,E), **leader** *t* and its BFS tree $G_{B}$ in Figure 3, we attempt to remove an arbitrary edge e=(u,v)∈E in the network and $d_{tv}\geq d_{tu}$. By observing the BFS tree $G_{B}$, we investigate that there are three edge deletion cases as follows:

- $d_{tu}=d_{tv}$: Since the ends of the removed edge *e*, *u* and *v* are at the same level of the bfs tree, it will not influence the shortest paths from *t* to all other nodes, i.e., $c_{t}\left(e\right)=c_{t}$.
- $d_{tv}>d_{tu}$ and ∃w∈N(v)$,d_{tw}=d_{tu}$: Assume that for s∈V, there exists a shortest path P=(t,…,u,v,…,s) in $G_{B}$. Since after removing the edge (u,v), there still exists a shortest path $P^{\prime }=\left(t,\ldots ,w,v,\ldots ,s\right)$ which has the same length, i.e., $d_{ts}\left(e\right)=d_{ts}$ as shown in Figure 3b. Hence, it will not influence the shortest paths from *t* to all other nodes, i.e., $c_{t}\left(e\right)=c_{t}$.
- $d_{tv}>d_{tu}$ and ∀w∈N(v)$,d_{tw}>d_{tu}$: Since after removing the edge (u,v), as shown in Figure 3c, $d_{tv}\left(e\right)=d_{tw}+1>d_{tv}$, so an update of the closeness value $c_{t}\left(e\right)$ is needed, specifically update the shortest paths of the affected nodes, *v* and its child nodes which have no neighbors in the upper level of the BFS tree (blacked out in Figure 3c).

#### 4.1.4. UpdateCloseness Algorithm

From the above observation, we have found that there are three cases after removing an edge in the network and only Case 3 needs to update the shortest paths of the affected node set. Due to such property, we propose the **UpdateCloseness** algorithm for the purpose of updating the closeness value of **leader** after removing an edge. The goal of our **UpdateCloseness** algorithm is to reduce the time cost of running a BFS, s.t., reduce the number of traversed edges and nodes when computing the sum of all shortest paths. The whole process of **UpdateCloseness** algorithm is summarized in Algorithm 2. In line 1–6, just similar to the Case 1 and 2 in Figure 3, if we find that the removed edge will not change the shortest paths from leader to all other nodes in the network, the algorithm will return the original closeness value and shortest paths array *d*.
**Algorithm 2:** UpdateCloseness.
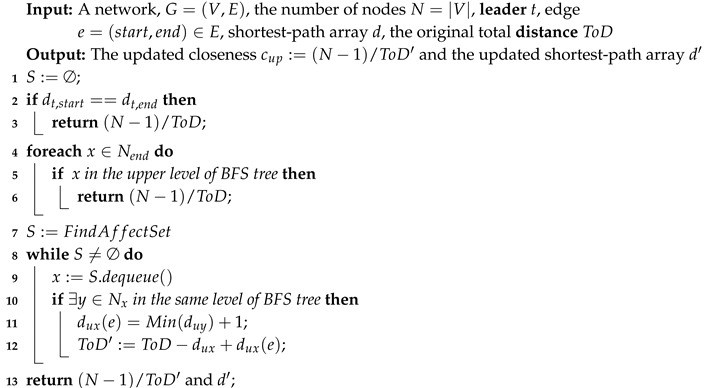


As mentioned in the example above, if the removed edge is similar to the Case 3, we must find the affected node set in the network. Therefore, we design an algorithm **FindAffectSet** to achieve this goal and the detail of this algorithm is shown in Algorithm 3. The result of Algorithm 3 is the affected node set *S*. In Algorithm 3, end is denoted as the node of the removed edge e∈E which is in the lower level of the BFS tree. The queue *Q* is designed to run a search in the child nodes of the node end to find the affected node set (lines 3–8). For each neighbor node of the node extracted from the queue *Q*, if there are no neighbors in the upper level of the BFS tree (line 7), it will be pushed into the queue *Q*. This search repeats until the queue *Q* is empty. By this pruning search, the affected node set *S* is found.
**Algorithm 3:** FindAffectSet.
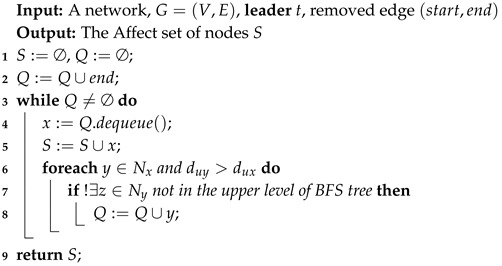


After finding the affected nodes set *S* (line 7 in Algorithm 2), we need to update the shortest paths from **leader** to all other affected nodes. For each node extracted from *S*, if it has neighbor nodes in the same level of the BFS tree, we update its distance from its neighbor node which is the nearest to **leader** *t* and update the total of all distances to *t* (lines 10–12 in Algorithm 2). This procedure will be repeated until all nodes in *S* have been updated and at last our **UpdateCloseness** algorithm will return the updated closeness value $c_{up}$ and the updated shortest paths array $d^{\prime }$. Intuitively, we can use this array $d^{\prime }$ in the next computation of the closeness value.

#### 4.1.5. Time Complexity Analysis

The time complexity of our pruning algorithm is analyzed in this section. In traditional way, after removing an edge, we must recompute the closeness value by BFS, which requires O(n+m). In our **UpdateCloseness** algorithm, we can reduce the traverse time by pruning the number of nodes that have to be traversed. In Case 1 or Case 2, the time complexity of the **UpdateCloseness** algorithm is only O(1). In Case 3, the number of the traversed nodes and edges is defined as $\tau_{nm}$, whose worst case is O(n+m); however, the worst case rarely happens, as shown in Section 5. We can find that the time complexity of our **UpdateCloseness** algorithm in Case 3 is $O(\tau_{nm})$ and therefore the greedy algorithm’s time complexity is $O(k\cdot m\cdot \tau_{nm})$. In most cases, we exploit that our **UpdateCloseness** algorithm can reduce plenty of time compared to BFS.

### 4.2. Approximation Algorithm for LCRMAX Problem (GSA)

In Section 3 we exploit that the optimization problem of closeness rank maximization is NP-hard. Hence, to obtain approximate solution in polynomial time, we consider a heuristic method combining greedy algorithm and simulated annealing algorithm (**GSA**). Algorithm 4 shows the detail of the heuristic algorithm. In the early work, we have found that the optimal solution in small-scale network is made up of neighbor nodes of **leader** which have higher degree. Hence, in Algorithm 4, we sort the neighbors of **leader**, i.e., N(u) in the descending order of degree (Line 1) so as to make the results obtained by greedy algorithm closer to the optimal solution. Due to the fact that we find that only greedy algorithm cannot obtain a $\left(1-\frac{1}{e}\right)$ approximation ratio like the value case, we exploit simulated annealing algorithm which takes greedy solution as an initial solution for the purpose of converging faster to a better solution in less time (line 9) and the detail of simulated annealing algorithm is shown in Algorithm 5.
**Algorithm 4:** Greedy and Simulated Annealing algorithm (GSA).
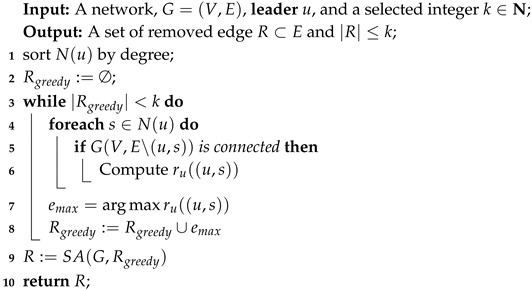

**Algorithm 5:** Simulated Annealing algorithm.
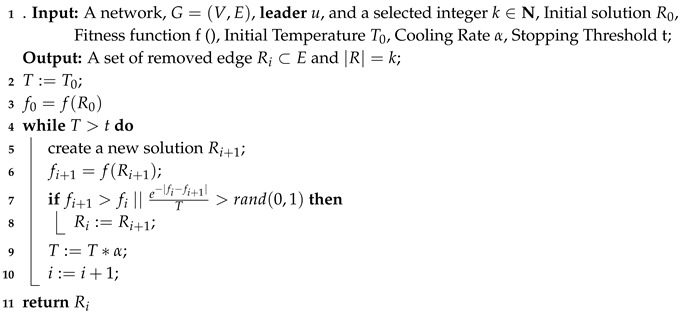


#### 4.2.1. The Reason for Proposing this Heuristic Method

Compared with the value approximation algorithm, we not only use the greedy algorithm to obtain the approximate solution due to the fact that the optimization problem in the closeness rank case is not **submodular** and there are situations where the gap between the approximate solution obtained by greedy algorithm and optimal solution is very huge. Table 2 shows a simple example to compare the closeness value case with the rank case.

In the closeness value case, generally, the closeness value decreases as the number of removed edges increases. Therefore, we can distinguish each edge by the closeness value result after removing it. Thus, the greedy algorithm works well in this case and we prove the **monotone** and **submodular** of this problem in Theorem 3. However, this does not apply in the rank case. In the network that **leader** has a huge advantage over other nodes in terms of closeness value (for example the scale-free network), when the size of the removed edges is less than 5, the probability of improving **leader**’s rank is very little, in other words, the rank of **leader** remains unchanged in most cases. Also, after removing a certain number of edges, the rank of **leader** can dramatically decrease, e.g., as shown in Table 2, after removing 5 edges, the **leader**’s rank varies from 1 to 6. In this example, the network has 17 nodes and **leader** has 15 neighbors, which satisfying the characteristics of scale-free network. In other words, except for one node, other nodes in the network have direct links with **leader**. Hence, the closeness value of **leader** is 0.94, which is close to the theoretical maximum of **closeness value**. The closeness values of other nodes in the network are mostly between 0.5 and 0.7, which are far less than **leader**. Therefore, we find that if the number of deleted edges is less than 5, then the closeness value of **leader** is still the largest in all the cases of edge deletion. In all cases, the minimum value of closeness is about 0.7, and the rank of **leader** is still 1, but the gap between **leader** and other nodes has been reduced. When five edges are deleted, the closeness value of **leader** drops to about 0.66 in the optimal case. At this time, there exist five nodes whose closeness value all around 0.69, so the optimal solution for deleting five edges is 6.

In this example, it is hard for the original greedy algorithm to obtain an approximation solution due to the fact that it cannot distinguish each edge if the rank remains unchanged. Hence, we consider combining the greedy algorithm with simulated annealing algorithm to make the approximate solution closer to the optimal solution.

#### 4.2.2. FastTopRank Algorithm

The heuristic algorithm proposed above has to compute the closeness rank of **leader** after removing an edge, which needs to solve the all-pairs-shortest-path (APSP) problem. The basic algorithm solving APSP problem is expensive, nearly O(n(n+m)) by running BFS for each node. Therefore, we propose a pruning algorithm, inspired by Bergamini’s top-*k* algorithm [7], to quickly compute the closeness rank of the given node. Compared to the **UpdateCloseness** algorithm, in this algorithm we attempt to reduce the times of BFS to compute the closeness rank.

The principle of our **FastTopRank** algorithm is to reduce the execution time of BFS when computing the rank of the top node for the reason that we would remove limited edges in the network and it is hard to dramatically reduce the top node’s rank. Differing from the Top-*k* algorithm [7], we assume that the value of *k* is equal to the number of nodes in the network, and we stop the algorithm when getting the accurate rank of the top node.

In Algorithm 6, first, we compute the lower bound of the sum of the shortest paths for all nodes v∈V by the two approaches proposed by Bergamini [7] (Line 3–4). The details of two lower bound algorithm are shown in Algorithms 7 and 8. Furthermore, in our algorithm, we design a priority queue *Q* to store all nodes order by increasing S(v). Please note that we choose the minimum of both lower bounds by level and neighbor (Line 5–8). To obtain the rank of the top node *u*, we extract a node with the minimum sum of the shortest paths $S^{*}$ from the head of *Q* (Line 11). If $S^{*}$ is just the lower bound (i.e., $visited[v^{*}]:=false$), Algorithm 6 will compute the exact value of the sum of shortest paths and insert it into *Q*. For the case that $S^{*}$ is the exact value of the sum of the shortest paths (i.e., $visited[v^{*}]:=true$), we can append this node and its $S^{*}$ to the top rank node list (Line 12–14). If fortunately we find the top node is in the top rank node list, we can sort the list by *S* and then obtain the accurate rank of the top node (Line 15–17).

Obviously, the lower bound close to the exact sum of the shortest paths can reduce the iterations to find the rank of the top node. We choose the minimum of two kinds of lower bounds which are proposed in Bergamini’s work [7] for this reason. Beyond that, the time complexity of our fast rank algorithm is $O(\left(n+m\right)\cdot k+klogk+\mu_{nm})$, where *k* is the number of iterations to find the accurate rank each of which needs to run BFS and klogk represents the time to sort the top rank note list. Also, $\mu_{nm}$ is the approximate time complexity of the two lower bound algorithms, whose exact complexity is based on the diameter of the network [7]. Despite that our algorithms may not be faster than the original algorithm when computing the node with low rank due to the reason that k≈n, s.t. the number of running BFS is nearly *n*. Our **FastTopRank** algorithm can significantly reduce the times of BFS, i.e., k≪n when asserting the rank of top nodes (the most cases of our optimization problem).
**Algorithm 6:** FastTopRank algorithm.
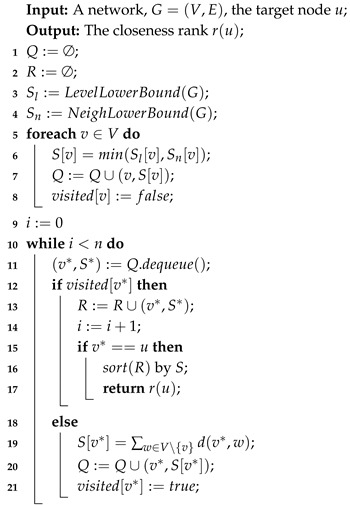

**Algorithm 7:** Level-based lower bound for undirected graphs
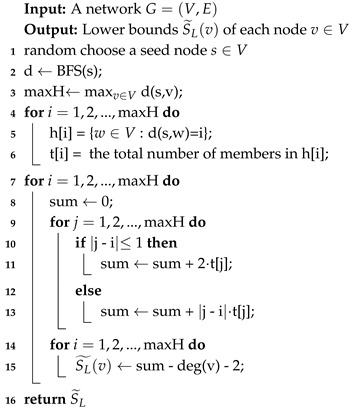

**Algorithm 8:** Neighborhood-based lower bound for undirected graphs
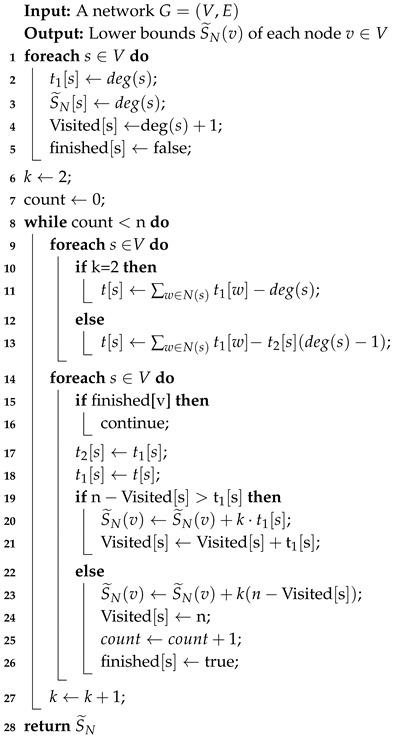


## 5. Experiment

In this section, we report the results of our experiments which examine the efficiency and accuracy of the proposed algorithms in Section 4. We implemented all algorithms in Python and ran all codes in a computer equipped with 16 GB memory and an Intel i5-8500 CPU (3.0 GHz) with 6 cores.

### 5.1. Dataset

To measure our algorithms, we have chosen some real-life networks and several random networks generated by different models. There are mainly three kinds of randomly generated networks as follows:

- Random network, which is generated by the Erdos–Renyi model [23]. The generated network can be denoted as G(n,p) with *n* nodes and *p* connection probability. This kind of network is denoted as ER.
- Small-world network, which is generated by the Watts–Strogatz model [24]. The generated graph can be denoted as G(n,k,p) with *n* nodes, *k* average degree and *p* rewiring edge probability. This kind of network is denoted as WS.
- Scale-free network, which is generated by the Barabási–Albert model [25]. The generated graph can be denoted as G(n,m) with *n* nodes and *m* edges to connect a new node with existing nodes. This kind of network is denoted as BA.

Normally, we generate corresponding size of random networks which are undirected and connected as needed and the specific size will be shown in each experiment. Moreover, we have selected some real-life networks and the detail of these networks is shown in Table 3. Some of the real-life networks are undirected and connected and the others are the networks transformed from the directed networks. All these networks are collected from the website of KONECT [26], SNAP [27] and Network Repository [28]. Due to the page limit, we only choose some typical results (3-4 networks) of some experiments.

### 5.2. Closeness Value Case Results

#### 5.2.1. Evaluate UpdateCloseness Algorithm

In this section, we evaluate the efficiency of our **UpdateCloseness** algorithm by comparing the computation time with BFS algorithm. To measure the efficiency of our algorithm, we proposed the notation of average speed up ratio *T* as follows:

(9) $$T=\frac{t_{BFS}}{t_{update}}$$

where $t_{BFS}$ and $t_{update}$ denote the average time cost by BFS algorithm and **UpdateCloseness** algorithm, respectively. We consider that the efficiency of the algorithm depends on the network size and the network topology and therefore, we conduct two different experiments to estimate the efficiency as follows:

- First, randomly generate networks in different size and kinds (BA, WS and ER). Then, randomly choose the node v∈V and a removed edge e∈E in the chosen network, then calculate the closeness value by BFS and **UpdateCloseness** for each time. We choose the average times by repeating it for 5000 times.
- First, choose some real-life complex networks as the datasets. Then, randomly choose the node v∈V and a removed edge e∈E in the chosen network, then calculate the closeness value by BFS and **UpdateCloseness** for each time. We choose the average times by repeating it for 5000 times.

Please note that in these two experiments, we assume that the closeness value has been computed before removing an edge and we estimate the efficiency when removing an edge. Also, there are three kinds of network size (≈$\frac{\left|E\right|}{\left|V\right|}$) of our randomly generated network, 5, 10, 15 and the node size varies from 100 to 5000.

**Figure 4:**
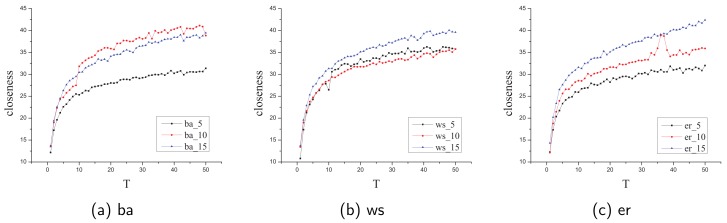
The average speed up ratio T in different sizes and kinds of random networks.

Figure 4 shows the average speed up ratio in different sizes and kinds of randomly generated networks. Regardless of the size and kind, our **UpdateCloseness** algorithm is always faster than the original BFS algorithm and the speed up ratio gradually increases as the network sizes (|V|) increases. In most cases, the speed up ratio tends to depend on $\frac{\left|E\right|}{\left|V\right|}$ and we confirm that our algorithm works well in complex networks.

Table 4 shows the average speed up ratio in the real-life complex networks. We find that our algorithm still works well in complex networks and the average speed up ratio T≈30. To summarize, the results of these experiments prove the efficiency of our **UpdateCloseness** algorithm in different networks, i.e., it works better than the original BFS algorithm in each case and it usually can achieve an speed up rate of more than ten in different kinds and sizes of networks.

#### 5.2.2. Compare Greedy Solution with the Optimal Solution

In this section, we estimate the accuracy of the approximation greedy algorithm by comparing its solution with the optimal solution in small-scale networks. Due to the limited computation resources, we select several real-life and randomly generated networks with dozens of nodes and edges as datasets and size of these networks is shown in Table 5.

Moreover, the budget of the removed edges *k* ranges from 1 to 5 (some to 7) and we proposed the notation of minimum approximation ratio (denoted by Min Appro Ratio) as the worst case in every budget *k* and it presents the ratio between the greedy solution and the optimal solution

Table 5 shows the Min Appro Ratio of each network and we discover that the worst case of our approximate greedy algorithm is far more accurate than the theorical lower bound ($1-\frac{1}{e}\approx 0.63$) which has been proved in Section 4.

**Figure 5:**
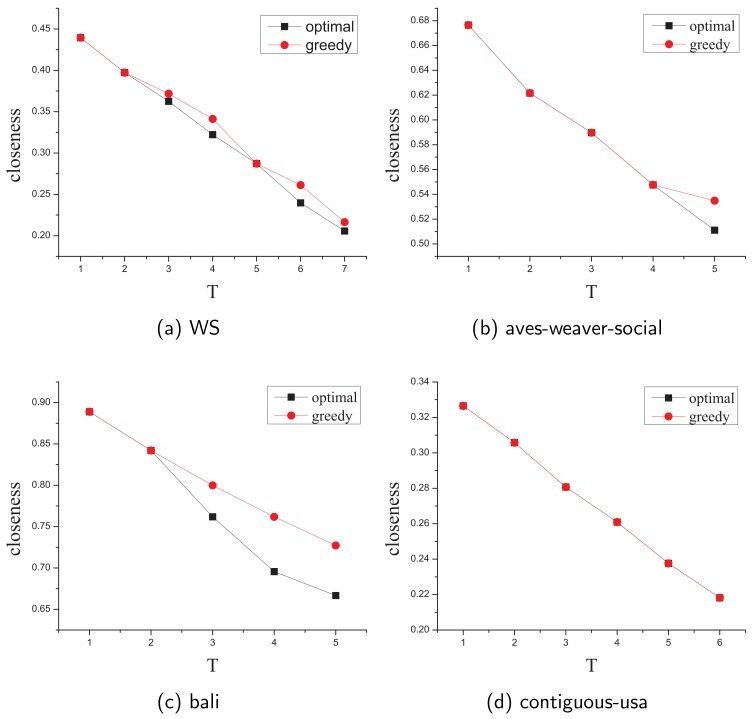
The comparison of the optimal and greedy closeness value results.

Figure 5 shows the differences between the optimal solution and greedy approximate solution in each budget *k* ( due to limited space, we only choose four typical results in all networks ). Obviously, our approximate greedy algorithm can significantly minimize the closeness value of the **leader** in seconds compared with nearly 2 days to obtain the optimal solution by brute-force search when the budget k=7.

#### 5.2.3. Compare Approximate Greedy Algorithm with Other Baseline Algorithms

In this section, we choose some baseline algorithms as comparison to evaluate the accuracy of our approximate greedy algorithm in the complex network. Different strategies of removing edges in the baseline algorithms are shown as follows:

- **Random:** randomly and uniformly select *k* edges in the whole network.
- **Top-*k* degree:** choose *k* edges with the highest degree sum.
- **Top-*k* closeness:** choose *k* edges with the highest closeness value sum.
- **Top-*k* neighbor degree:** choose *k* edges in the neighbor of the **leader** node with the highest degree.

Please note that the first baseline algorithm is easy to understand and can be implement within a short time. Furthermore, the fourth baseline algorithm is a kind of variety of the ROAM algorithm in Waniek’s work [3]. Table 6 shows the detail of all real-life and randomly generated complex networks used in this comparison experiment.

**Figure 6:**
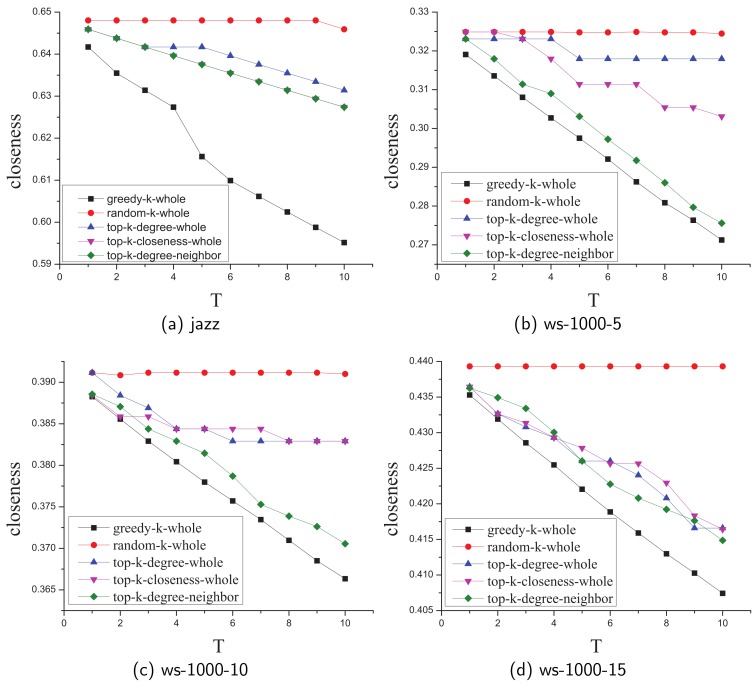
The comparison of the greedy algorithm with the baseline algorithms.

Figure 6 shows the different results in random networks or real-life networks ( due to limited space, we only choose four typical results in all networks ). We observe that our greedy algorithm works better than other four comparison algorithms in general and the three top-k algorithms can only obtain lower approximation results. **Random** algorithm performs poorly in all networks because there are limited edges whose deletion can contribute to closeness decrement in complex networks.

### 5.3. Closeness Rank Case Results

#### 5.3.1. Evaluate FastTopRank Algorithm

In this section, similar to the experiment in the closeness value case, we conduct an experiment to evaluate the efficiency of the **FastTopRank** algorithm. First, due to the fact that our algorithm can compute the exact closeness rank of the node in the top rank rapidly, we choose to speed up rate of the nodes with top-50 rank as the standard of this experiment. Furthermore, we denote the notation of speed up rate $T_{rank}$ as follows:

(10) $$T_{rank}=\frac{T_{whole}}{T_{partial}}$$

where $T_{partial}$ and $T_{whole}$ denote the time cost of **FastTopRank** and traditional algorithm (computing the closeness value of all nodes and sorting them).

**Figure 7:**
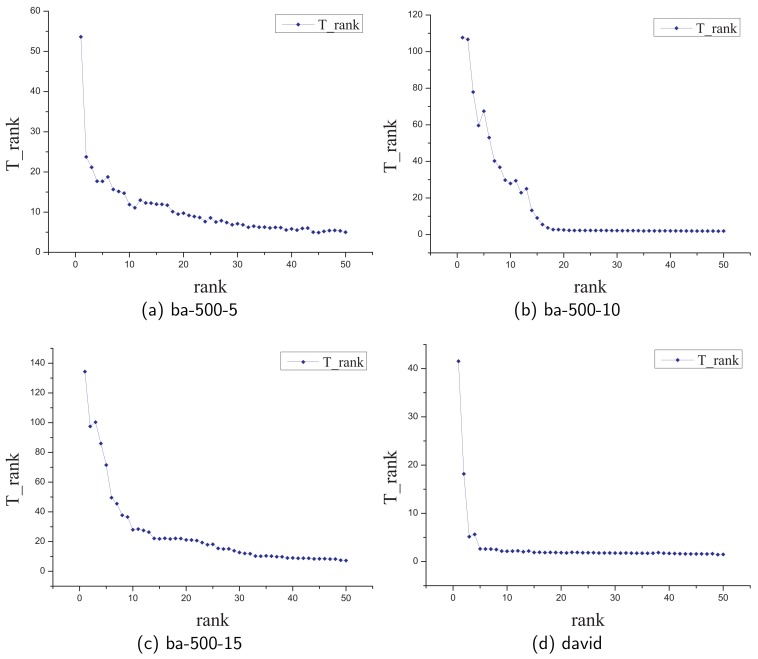
Evaluate the efficiency of the **FastTopRank** algorithm.

In this experiment, we compute the rank of the nodes ranking from 1 to 50 in the network by two algorithms for three times and take the average to compute the speed up rate $T_{rank}$. Figure 7 shows the results in different networks. Obviously, our algorithm can significantly reduce the time when computing the top-5 nodes and it still works when computing the nodes with low rank.

#### 5.3.2. Compare the Solution of GSA Algorithm with the Optimal Solution

In this experiment, we test our GSA algorithm in some small-scale networks and compare its solution with the optimal solution obtained by brute-force search. The networks of this experiment are shown in Table 5 and results are shown in Table 7. Due to the limitation of computation resources, the budget of the removed edges only ranges from 1 to 5 and we just choose three typical results due to the page limit.

In most cases, the solution of our GSA algorithm is the same or very close to the optimal solution. However, there are still some situations where the approximate solution is far from the optimal solution in the network such as BA network whose **leader**’s degree is particularly high and we find that its optimal solution, i.e., the closeness rank of **leader** remains the first until removing as many edges as possible. Hence, in this situation, the number of optimal solutions is few and it is hard for our algorithm to obtain an approximate solution.

#### 5.3.3. Compare GSA Algorithm with Other Baseline Algorithms

In this section, to estimate the accuracy of our GSA algorithm in the complex network, we compare our algorithm with the following baseline algorithms:

- **Greedy Neighbor:** the greedy algorithm that chooses the neighbor edges that maximize the closeness rank each time.
- **Top-*k* degree:** choose *k* edges with the highest degree sum.
- **Top-*k* closeness:** choose *k* edges with the highest closeness value sum.
- **Top-*k* neighbor degree:** choose *k* edges in the neighbor of the **leader** node with the highest degree.

In the earlier experiment, we have found that the optimal solution consists of neighbor edges in most cases, and removing limited edges cannot change the closeness rank of **leader**. Therefore, differing from the comparison with the optimal solution, in this experiment we set the range of the budget of removing edges at 10–50% of the degree of the **leader** to protect the integrity of network as much as possible and make the effect of improving the closeness rank more obvious. Furthermore, in general, we assume that the network remains connected after removing the edge set generated by base line algorithms.

Table 8, Table 9 and Table 10 show the results of this experiment. We find that the GSA algorithm can improve the results obtained by the greedy neighbor algorithms and the other three baseline algorithms perform poorly in all networks. Furthermore, we find that removing limited edges in the WS and ER networks can significantly increase the rank of the **leader**.

## 6. Conclusions

We present two optimization problems of hiding *leader* in the IoT networks by minimizing (maximizing) closeness value (rank). We show finding optimal solutions is NP-hard. Hence, we propose two heuristic algorithms to solve optimization problems in polynomial time and prove that the greedy algorithm can obtain a $\left(1-\frac{1}{e}\right)$-approximation ratio. We also provide two pruning algorithms to reduce the time cost of BFS algorithm and compute the closeness value of each nodes in the complex networks. Experimental results show that our pruning algorithms can reduce the time by at least 10 times to calculate the closeness value (rank) compared with original algorithms and our heuristic algorithms can be close to the optimal solutions and outperform the solutions by the baseline algorithms.

In the future, we would like to combine our work with existing studies of privacy preservation in IoT focus on access control model, such as context-aware access control model [30,31,32,33,34], to ensure the privacy and security of IoT networks. We hope to construct a context-aware access control policy framework combining the idea of our dynamic update algorithm and can dynamic update the access control policy in limited time.

## Figures and Tables

**Table 1:** Comparison with related works.

Scheme	Edges Updated	Measurement	Selection Range	Hidden Effect	Solution Goal
Waniek [3]	Addition and Deletion	Degree Centrality [18]	Neighbors	Yes	Value
Crescenzi [19]	Addition	Harmonic Centrality [20]	Neighbors	No	Value
Our work	Deletion	Closeness Centrality [4]	Entire Network	Yes	Value and Rank

**Table 2:** The simple example to compare the closeness value cases with the rank cases.

Delete Edges	Closeness Value (Optimal)	Closeness Rank (Optimal)	Closeness Rank (Greedy)
1	0.89	1	1
2	0.84	1	1
3	0.76	1	1
4	0.69	1	1
5	0.66	**6**	1

**Table 3:** Real-life datasets.

Network	|V|	|E|	Network Type
WTC [29]	36	64	Terrorist Network
bali	17	63	Terrorist Network
moreno-rhesus	16	69	Animal Social Network
aves-weaver-social	24	62	Animal Social Network
Dolphins	62	159	Animal Social Network
ContiguousUSA	49	107	Infrastructure Network
david	112	425	Lexical Network
Jazz	198	2742	Collaboration Network
arenas-email	1133	5451	Communication Network
arenas-pgp	10,680	24,316	Interaction Network
as-caida	26,475	106,762	Internet Network
ucidata-gama	16	58	Social Network
moreno-taro	22	39	Social Network
moreno-beach	37	105	Social Network
moreno-oz	217	1839	Social Network
FB-tvshow	3892	17,262	Social Network
FB-politician	5908	41,729	Social Network
FB-government	7057	89,455	Social Network

**Table 4:** Complex network datasets used in evaluating the efficiency of our **UpdateCloseness** algorithm.

Network	|V|	|E|	Speed up Ratio
arenas-email	1133	5451	26.52
FB-tvshow	3892	17,262	28.95
FB-politician	5908	41,729	32.59
FB-government	7057	89,455	36.15
arenas-pgp	10,680	24,316	32.24
as-caida	26,475	106,762	31.46

**Table 5:** Datasets used in comparing greedy solutions with the optimal solutions.

Network	|V|	|E|	Min Appro Ratio
WTC	36	64	0.9632
bali	17	63	0.9130
aves-weaver-social	24	62	0.9556
moreno-rhesus	16	69	0.9200
moreno-beach	37	105	1.0000
moreno-taro	22	39	0.8852
dolphins	62	159	0.9600
contiguous-usa	49	107	1.0000
ucidata-gama	16	58	0.9231
Random graph	30	55	0.8511
Scale-free	30	56	0.9437
Small-world	30	60	0.9174

**Table 6:** Datasets used in comparing greedy solution with the other algorithms.

Network	|V|	|E|
Jazz	198	2742
moreno-oz	217	1839
david	112	425
FB-tvshow	3892	17,262
FB-politician	5908	41,729
arenas-email	1133	5451
BA-5	1100	5475
BA-10	1000	9900
BA-15	1000	14,775
ER-5	1000	5025
ER-10	1000	10,029
ER-15	1000	14,917
WS-5	1000	5000
WS-10	1100	10,000
WS-15	1100	15,000

**Table 7:** Compare the optimal solution with the approximate solutions.

	Aves-Weaver-Social			Dolphins			Moreno_Rhesus		
**k**	**Optimal**	**Greedy**	**GSA**	**Optimal**	**Greedy**	**GSA**	**Optimal**	**Greedy**	**GSA**
1	1	1	1	3	3	3	1	1	1
2	1	1	1	10	6	10	6	5	6
3	1	1	1	23	17	23	10	8	10
4	1	1	1	36	36	36	10	10	10
5	5	1	5	50	50	50	13	11	13

**Table 8:** The comparison of the approximate algorithm with the baseline algorithms: moreno-oz.

k	Greedy	GSA	Top-*k*-Degree	Top-*k*-Closeness	Top-*k*-Neighbor
5	2	2	1	1	1
6	2	2	1	1	1
7	2	2	1	1	1
8	3	4	1	1	1
9	5	6	1	1	1
10	5	6	1	1	2
11	5	6	1	1	2
12	6	8	1	1	2
13	8	9	1	1	2
14	9	9	1	1	2
15	10	10	1	1	2
16	13	20	1	1	3
17	17	19	1	1	5
18	19	23	1	1	5
19	22	25	1	1	5
20	24	32	1	1	5
21	30	35	1	1	5
22	35	39	1	1	5
23	38	39	1	1	8
24	46	52	2	1	10
25	52	54	2	1	10
26	54	62	3	1	11
27	63	65	3	1	19
28	67	73	3	1	20

**Table 9:** The comparison of the approximate algorithm with the baseline algorithms: er-150.

k	Greedy	GSA	Top-*k*-Degree	Top-*k*-Closeness	Top-*k*-Neighbor
3	2	6	1	1	2
4	6	6	2	2	6
5	12	22	6	6	6
6	30	30	11	11	11
7	33	33	20	20	21
8	42	42	30	30	29
9	61	61	33	33	33
10	70	94	42	42	42
11	94	111	42	52	52
12	111	111	42	61	70
13	119	124	52	78	78
14	122	128	61	77	95
15	140	140	78	77	111
16	140	143	77	77	118
17	144	144	77	77	124
18	145	146	77	77	135
19	148	148	77	77	138

**Table 10:** The comparison of the approximate algorithm with the baseline algorithms: er-200.

k	Greedy	GSA	Top-*k*-Degree	Top-*k*-Closeness	Top-*k*-Neighbor
4	6	8	1	1	1
5	10	10	1	1	2
6	11	11	1	1	6
7	14	19	1	2	6
8	26	36	1	1	9
9	35	37	1	6	11
10	40	40	1	10	17
11	54	60	4	16	33
12	65	77	10	16	37
13	89	104	11	16	47
14	88	104	11	24	55
15	111	118	10	24	65
16	129	156	26	37	77
17	162	177	33	40	88
18	177	181	33	55	112
19	184	186	33	65	141
20	189	192	37	73	156
21	195	197	47	73	179
